# Dominant mutations in the severe acute respiratory syndrome coronavirus‐2 genome challenge polymerase chain reaction detection

**DOI:** 10.1002/ctd2.23

**Published:** 2022-01-18

**Authors:** Teng Liu, Leshan Xiu, Kun Yin, Shengli Li

**Affiliations:** ^1^ Precision Research Center for Refractory Diseases Institute for Clinical Research Shanghai General Hospital Institute for Clinical Research Shanghai Jiao Tong University School of Medicine Shanghai 201620 China; ^2^ School of Global Health Chinese Centre for Tropical Diseases Research Shanghai Jiao Tong University School of Medicine Shanghai China

Dear Editor,

Severe acute respiratory syndrome coronavirus‐2 (SARS‐CoV‐2) caused the new coronavirus disease‐2019 (COVID‐19) pandemic that began in late 2019 and has caused more than 225 million infections and 4.6 million deaths worldwide. SARS‐CoV‐2 is a Betacoronavirus similar to SARS‐CoV‐1 that led to the 2003 epidemic and the Middle East respiratory syndrome–coronavirus that was first reported in Saudi Arabia in 2012. SARS‐CoV‐2, as with other coronaviruses, has a 30k base‐pair linear genome with 10 open reading frames,^1^, ^2^ including ORF1ab, spike protein (S), envelope protein (E), membrane protein (M), nucleocapsid protein (N), ORF3a, ORF6, ORF7ab, ORF8 and ORF10. Reverse transcription‐quantitative polymerase chain reaction (RT‐qPCR) has become one of the gold standards for the diagnosis of SARS‐CoV‐2 infection.^3^ Most RT‐qPCR primers/probes are designed for the ORF1ab, E or N genes due to their high conservation among sarbecoviruses.^4^ However, as an RNA virus, SARS‐CoV‐2 has a relatively high mutation rate, and several dominant mutations have emerged, such as Alpha (B.1.1.7), Beta (B.1.351), Gamma (P1) and Delta (B.1.617).^5^ The sensitivity of RT‐qPCR detection kits is largely affected when SARS‐CoV‐2 genome sequences that are covered in detecting primers/probes are mutated.^6^ Some cases are false‐negative during regular quarantine but positive a few weeks after quarantine.^7^ These missing cases may be caused by mismatches in detecting primers/probes.

To identify mismatches in SARS‐CoV‐2 detection primers/probes, we downloaded the latest 500 000 full‐length sequences of SARS‐CoV‐2 genomes from GISAID (https://www.gisaid.org/) that were uploaded up to 10 November 2021. We obtained 499 481 sequences for further analysis after quality control (Figure 1A). Ninety primer/probe sequences of 21 RT‐qPCR kits were retrieved from the World Health Organization website (https://www.who.int/), the Center for Disease Control and Prevention of China (https://www.chinacdc.cn/), the United States (https://www.cdc.gov/), Japan (https://www.niid.go.jp/), Charité–Universitätsmedizin Berlin of Germany (https://www.Charité.de/), Institute Pasteur of France (https://www.pasteur.fr/), The University of Hong Kong (HKU) and recent publications^4^, ^8^ (Figure 1A and Table S1). Then, we aligned these primer/probe sequences to the 499 481 SARS‐CoV‐2 genomes and summarized the mismatches (Figure 1A and Table S1). BLASTn software was applied to perform the alignment with the following parameters: *blastn ‐task blastn‐short ‐db SARS‐CoV‐2_full‐length_gemome_sequences_db_file ‐outfmt 7 ‐query primer_probe_RT‐qPCR_fasta_file ‐out result_file ‐max_target_seqs 500 000*. In‐house scripts were used to check and correct the original alignment results. Then, the mismatched bases of primers/probes were identified across these SARS‐CoV‐2 genome sequences (Table S1). SARS‐CoV‐2 lineage identification and cumulative case statistics were retrieved from Cov‐Lineage.org.^9^ PerlPrimer was employed to predict the melting temperature of the original and suggested primers/probes.^10^

**FIGURE 1:**
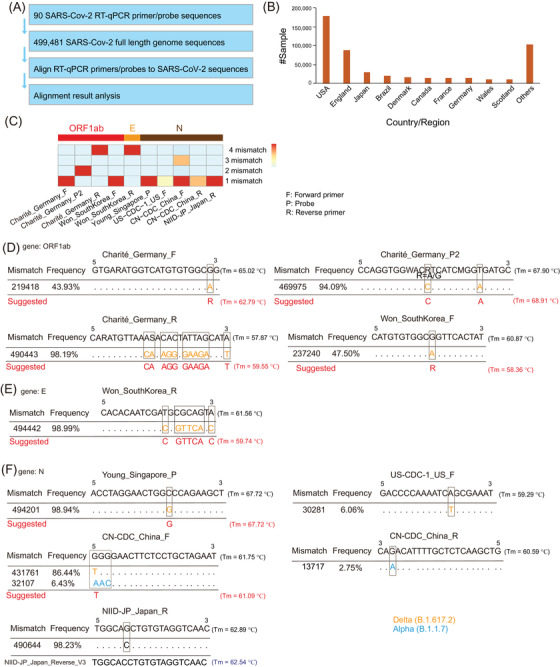
Identification of mismatches between the severe acute respiratory syndrome coronavirus‐2 (SARS‐CoV‐2) genome and detection primers/probes. (A) Overview of the study design. (B) The country/region distribution of uploaded SARS‐CoV‐2 sequence samples. (C) Heatmap shows top mismatches primer/probe sequences. (D) Detailed information on primer/probe sequences in the ORF1ab gene. (E) Detailed information on primer/probe sequences in the E gene. (F) Detailed information on primer/probe sequences in gene N. *T*_m_: melting temperature

Among these primers/probes, 32, 11, 14 and 33 were located in the ORF1ab, S, E and N genes, respectively (Table S1). Most primers/probes had at least one mismatch and some even had more than four mismatches (Table S1). More than half of the 499 481 SARS‐CoV‐2 genome sequences were from the United States and England. Primer/probe sequences from Charité‐Universitätsmedizin Berlin and the Won group of South Korea, which were located in ORF1ab, had very high mismatch rates (Figure 1C). The reverse primer of the Won group of South Korea had a high mismatch rate (Figure 1C). Five sequences from the Young lab from Singapore, the Centers for Disease Control and Prevention (CDC) of the United States, CDC of China, and the National Institute of Infectious Diseases (NIID) of Japan had high mismatch rates in the N gene (Figure 1C).

The top unmatched sequences and the suggested improvement are displayed in Figure 1D–F. The suggested bases were presented based on the mismatch frequencies. Degenerate bases are suggested when two or more bases exist. The mismatch frequency of the forward primer of Charité‐Universitätsmedizin Berlin in ORF1ab was 43.9% (Figure 1D), and we suggested a degenerate base R to replace the original G, and the predicted melting temperature (*T*_m_) was changed from 65.02 to 62.79°C. The probe2 and reverse primer mismatch frequencies of Charité–Universitätsmedizin Berlin in ORF1ab were 94.09% and 98.19%, respectively. We suggested changing the original base to the dominant mutations (Figure 1D). The forward primer mismatch frequency of the Won group was 47.5% in ORF1ab, and a degenerate base R was suggested (Figure 1D). Seven bases of the E gene were mismatched in the reverse primer of the Won group in 98.99% of sequences, and we suggested changing these seven bases (Figure 1E). The probe sequence mismatch frequency was 98.94%, and we suggested using G to replace the original C, and the *T*_m_ was not changed (Figure 1F). The forward primer mismatch frequency of US CDC‐1 was 6.06%, and the reverse primer mismatch frequency of the China CDC was 2.75%, the original bases of which were still useful. The frequencies of two mismatches from the China CDC forward primer in the N gene were 86.44% and 6.43% (Figure 1F), and we suggested *T* to replace the original G with the *T*_m_ changed from 61.75 to 61.09°C. The v3 reverse sequences of NIID‐JP have changed original G to C to correct the mismatch (Figure 1F). The mismatches of Charité–Universitätsmediz in Berlin forward/reverse primers in ORF1ab (Figure 1D) and Won group reverse primer in the E gene occurred at the 3′ of the primer sequences, which may have a strong influence on the detection sensitivity because the PCR replication starts on 3′ of the template primers/probes.

In conclusion, rapid mutation of the SARS‐CoV‐2 genome has largely challenged the PCR detection of infected populations with mutated SARS‐CoV‐2. Missing these infected populations, especially those in international trips and regular quarantine, has caused a series of community spreads. Identification and renewal of mismatches in current detection kits will help with the timely diagnosis of SARS‐CoV‐2 infection and the control of epidemic spread. Herein, we analysed the mismatches between diagnostic PCR assays and the latest SARS‐CoV‐2 genomes and found that 10 out of 90 primers/probes had high mismatch frequencies. We also suggested renewal strategies. Web‐based tools, such as NextClade and PrimerChecker of GISAID, are available to evaluate the mismatches of SARS‐CoV‐2 RT‐PCR primers/probes, which is very helpful for checking and renewing primers/probes. Based on the cumulative case statistics (Figure S1), it is necessary to check PCR primers/probes in 3–6 months to avoid mismatches.

## FUNDING INFORMATION

The funding name is Shanghai General Hospital Startup Funding. The funding numbers are 02.06.01.20.06 and 02.06.02.21.01.

## CONFLICT OF INTEREST

The authors declare that they have no conflict of interest.

## Supporting information

FigureS1

TableS1
