# Supplementary figures and images for: Dominant mutations in the severe acute respiratory syndrome coronavirus‐2 genome challenge polymerase chain reaction detection
Source: Clin Transl Discov. 2022 Jan 18;2(1):e23. doi: 10.1002/ctd2.23 (PMC9015413; doi:10.1002/ctd2.23)

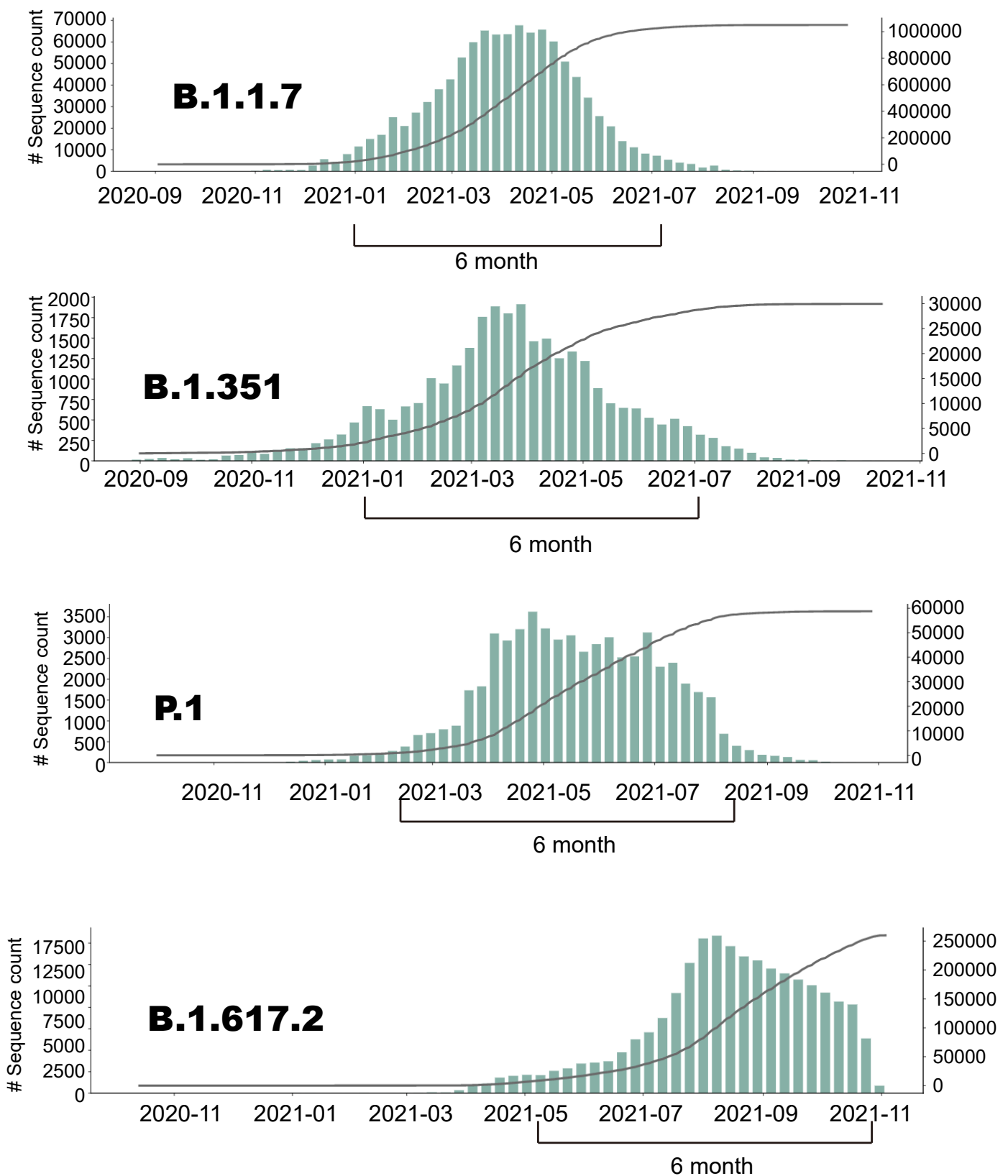

**Supplemental Figure S1.** The cumulative case statistics of four major SARS-CoV-2 lineages in 2021.

Supplement: Supplementary file 1 — FigureS1 [file CTD2-2-0-s001.pdf]
