# Supplementary material for: Dominant mutations in the severe acute respiratory syndrome coronavirus‐2 genome challenge polymerase chain reaction detection
Source: Clin Transl Discov. 2022 Jan 18;2(1):e23. doi: 10.1002/ctd2.23 (PMC9015413; doi:10.1002/ctd2.23)
Supplement: Supplementary file 2 — TableS1 [file CTD2-2-0-s002.docx]

**Supplemental Table S1. Summary of mismatches between RT-qPCR primers/probes and SARS-CoV-2 genome sequences.**

| Gene target | Source | Country/Region | F/P/R* | Sequence (5'-3') | Nucleotide position | Loss Hits | Hits | Perfect match | 1 mismatch | 2 mismatches | 3 mismatches | ≥ 4 mismatches | total unmatched |
| --- | --- | --- | --- | --- | --- | --- | --- | --- | --- | --- | --- | --- | --- |
| ORF1AB | Yip | China | F | ATGCATTTGCATCAGAGGCT | 1866–>1885 | 2253 | 497228 | 494451 | 2777 | 0 | 0 | 0 | 5030 |
|  | Yip | China | R | TTGTTATAGCGGCCTTCTGT | 1970<–1951 | 4401 | 495080 | 492555 | 2524 | 1 | 0 | 0 | 6926 |
|  | Pasteur-1 | France | F | ATGAGCTTAGTCCTGTTG | 12690–>12707 | 1643 | 497838 | 497635 | 203 | 0 | 0 | 0 | 1846 |
|  | Pasteur-1 | France | P | AGATGTCTTGTGCTGCCGGTA | 12717–>12737 | 1741 | 497740 | 494350 | 621 | 2769 | 0 | 0 | 5131 |
|  | Pasteur-1 | France | R | CTCCCTTTGTTGTGTTGT | 12797<–12780 | 6780 | 492701 | 491474 | 1227 | 0 | 0 | 0 | 8007 |
|  | Pasteur-2 | France | F | GGTAACTGGTATGATTTCG | 14080–>14098 | 1009 | 498472 | 498437 | 35 | 0 | 0 | 0 | 1044 |
|  | Pasteur-2 | France | P | TCATACAAACCACGCCAGG | 14105–>14123 | 6540 | 492941 | 492793 | 148 | 0 | 0 | 0 | 6688 |
|  | Pasteur-2 | France | R | CTGGTCAAGGTTAATATAGG | 14186<–14167 | 901 | 498580 | 497190 | 1390 | 0 | 0 | 0 | 2291 |
|  | CN-CDC | China | F | CCCTGTGGGTTTTACACTTAA | 13342–>13362 | 4193 | 495288 | 494197 | 1090 | 1 | 0 | 0 | 5284 |
|  | CN-CDC | China | P | CCGTCTGCGGTATGTGGAAAGGTTATGG | 13377–>13404 | 12756 | 486725 | 485336 | 1238 | 99 | 52 | 0 | 14145 |
|  | CN-CDC | China | R | ACGATTGTGCATCAGCTGA | 13460<–13442 | 2643 | 496838 | 496765 | 73 | 0 | 0 | 0 | 2716 |
|  | Young | Singapore | F | TCATTGTTAATGCCTATATTAACC | 14155–>14178 | 864 | 498617 | 498374 | 240 | 3 | 0 | 0 | 1107 |
|  | Young | Singapore | P | AACTGCAGAGTCACATGTTGACA | 14193–>14215 | 1255 | 498226 | 496718 | 1505 | 3 | 0 | 0 | 2763 |
|  | Young | Singapore | R | CACTTAATGTAAGGCTTTGTTAAG | 14243<–14220 | 821 | 498660 | 497903 | 756 | 1 | 0 | 0 | 1578 |
|  | Charité | Germany | F | GTGARATGGTCATGTGTGGCGG | 15431–>15452 | 1701 | 497780 | 278199 | 219418 | 163 | 0 | 0 | 221282 |
|  | Charité | Germany | P1 | CAGGTGGAACCTCATCAGGAGATGC | 15470–>15494 | 8787 | 490694 | 489997 | 692 | 5 | 0 | 0 | 9484 |
|  | Charité | Germany | P2 | CCAGGTGGWACRTCATCMGGTGATGC | 15469–>15494 | 29337 | 470144 | 0 | 0 | 469975 | 0 | 169 | 499481 |
|  | Charité | Germany | R | CARATGTTAAASACACTATTAGCATA | 15530<–15505 | 8424 | 491057 | 0 | 614 | 0 | 0 | 490443 | 499481 |
|  | Won | South Korea | F | CATGTGTGGCGGTTCACTAT | 15441–>15460 | 9987 | 489494 | 252253 | 237240 | 1 | 0 | 0 | 247228 |
|  | Won | South Korea | R | TGCATTAACATTGGCCGTGA | 15558<-15539 | 3192 | 496289 | 495114 | 1175 | 0 | 0 | 0 | 4367 |
|  | Chan | China | F | CGCATACAGTCTTRCAGGCT | 16220–>16239 | 2949 | 496532 | 496523 | 3 | 6 | 0 | 0 | 2958 |
|  | Chan | China | P | TTAAGATGTGGTGCTTGCATACGTAGAC | 16276–>16303 | 9975 | 489506 | 483990 | 5354 | 132 | 30 | 0 | 15491 |
|  | Chan | China | R | GTGTGATGTTGAWATGACATGGTC | 16352<–16330 | 10996 | 488485 | 484117 | 0 | 4367 | 1 | 0 | 15364 |
|  | Wu | China | F | TGATGATACTCTCTGACGATGCTGT | 15704->15728 | 776 | 498705 | 491394 | 7277 | 34 | 0 | 0 | 8087 |
|  | Wu | China | P | ATGCATCTCAAGGTCTAGTG | 15749->15768 | 818 | 498663 | 496410 | 2253 | 0 | 0 | 0 | 3071 |
|  | Wu | China | R | CTCAGTCCAACATTTTGCTTCAGA | 15846->15823 | 333 | 499148 | 498844 | 302 | 2 | 0 | 0 | 637 |
|  | HKU | Hong Kong | F | TGGGGYTTTACRGGTAACCT | 18778–>18797 | 2193 | 497288 | 497287 | 0 | 0 | 1 | 0 | 2194 |
|  | HKU | Hong Kong | P | TAGTTGTGATGCWATCATGACTAG | 18849–>18872 | 888 | 498593 | 497916 | 0 | 677 | 0 | 0 | 1565 |
|  | HKU | Hong Kong | R | AACRCGCTTAACAAAGCACTC | 18909<–18889 | 2548 | 496933 | 493954 | 2953 | 26 | 0 | 0 | 5527 |
|  | Lu | China | F | AGAAGATTGGTTAGATGATGATAGT | 3193->3217 | 1697 | 497784 | 496763 | 1003 | 18 | 0 | 0 | 2718 |
|  | Lu | China | P | TCCTCACTGCCGTCTTGTTGACCA | 3252<-3229 | 1906 | 497575 | 495711 | 1848 | 16 | 0 | 0 | 3770 |
|  | Lu | China | R | TTCCATCTCTAATTGAGGTTGAACC | 3310<-3286 | 1840 | 497641 | 495477 | 2150 | 14 | 0 | 0 | 4004 |
| S | Young | Singapore | F | TATACATGTCTCTGGGACCA | 21763–>21782 | 68108 | 431373 | 425933 | 5439 | 1 | 0 | 0 | 73548 |
|  | Young | Singapore | P | CTAAGAGGTTTGATAACCCTGTCCTACC | 21789–>21816 | 53657 | 445824 | 435402 | 9709 | 640 | 73 | 0 | 64079 |
|  | Young | Singapore | R | ATCCAGCCTCTTATTATGTTAGAC | 21876<–21853 | 56495 | 442986 | 440654 | 2235 | 96 | 1 | 0 | 58827 |
|  | Chan | China | F | CCTACTAAATTAAATGATCTCTGCTTTACT | 22712–>22741 | 258 | 499223 | 498480 | 711 | 11 | 17 | 4 | 1001 |
|  | Chan | China | P | CGCTCCAGGGCAAACTGGAAAG | 22792–>22813 | 1146 | 498335 | 490933 | 7402 | 0 | 0 | 0 | 8548 |
|  | Chan | China | R | CAAGCTATAACGCAGCCTGTA | 22869<–-22849 | 18671 | 486523 | 479361 | 1449 | 0 | 0 | 0 | 20120 |
|  | Won | South Korea | F | CTACATGCACCAGCAACTGT | 23114->23133 | 14782 | 484699 | 482449 | 2249 | 1 | 0 | 0 | 17032 |
|  | Won | South Korea | R | CACCTGTGCCTGTTAAACCA | 23213<–23194 | 10174 | 489307 | 488123 | 1184 | 0 | 0 | 0 | 11358 |
|  | Wei | US | F | TCAACTCAGGACTTGTTCTTAC | 21710->21731 | 34519 | 464962 | 460163 | 4795 | 4 | 0 | 0 | 39318 |
|  | Wei | US | P | TGGTCCCAGAGACATGTATAGCAT | 21796->21817 | 67717 | 431764 | 425809 | 5866 | 88 | 1 | 0 | 73672 |
|  | Wei | US | R | TGGTAGGACAGGGTTATCAAAC | 21759->21782 | 54771 | 444710 | 436357 | 8349 | 4 | 0 | 0 | 63124 |
| E | Won | South Korea | F | TTCGGAAGAGACAGGTACGTT | 26259–>26279 | 1524 | 497957 | 497226 | 731 | 0 | 0 | 0 | 2255 |
|  | Won | South Korea | R | CACACAATCGATGCGCAGTA | 26365<–26346 | 5033 | 494448 | 0 | 6 | 0 | 0 | 494442 | 499481 |
|  | Pasteur | France | F | ACAGGTACGTTAATAGTTAATAGCGT | 26269–>26294 | 1524 | 497957 | 497281 | 631 | 37 | 8 | 0 | 2200 |
|  | Pasteur | France | P | ACACTAGCCATCCTTACTGCGCTTCG | 26332–>26357 | 3322 | 496159 | 494704 | 1393 | 52 | 10 | 0 | 4777 |
|  | Pasteur | France | R | ATATTGCAGCAGTACGCACACA | 26381<–26360 | 4185 | 495296 | 494583 | 711 | 2 | 0 | 0 | 4898 |
|  | Huang | China | F | ACTTCTTTTTCTTGCTTTCGTGGT | 26295–>26318 | 2860 | 496621 | 495031 | 1567 | 23 | 0 | 0 | 4450 |
|  | Huang | China | P | CTAGTTACACTAGCCATCCTTACTGC | 26326–>26351 | 3258 | 496223 | 494770 | 1387 | 59 | 7 | 0 | 4711 |
|  | Huang | China | R | GCAGCAGTACGCACACAATC | 26376<–26357 | 4251 | 495230 | 494591 | 630 | 9 | 0 | 0 | 4890 |
|  | Niu | China | F | TTCTTGCTTTCGTGGTATTC | 26303–>26322 | 3238 | 496243 | 495365 | 878 | 0 | 0 | 0 | 4116 |
|  | Niu | China | P | GTTACACTAGCCATCCTTACTGCGCTTCGA | 26329–>26358 | 3201 | 496280 | 494611 | 1587 | 63 | 17 | 2 | 4870 |
|  | Niu | China | R | CACGTTAACAATATTGCAGC | 26391<–26372 | 4140 | 495341 | 495132 | 209 | 0 | 0 | 0 | 4349 |
|  | Charité | Germany | F | ACAGGTACGTTAATAGTTAATAGCGT | 26269–>26294 | 1524 | 497957 | 497281 | 631 | 37 | 8 | 0 | 2200 |
|  | Charité | Germany | P | ACACTAGCCATCCTTACTGCGCTTCG | 26298->26323 | 3322 | 496159 | 494704 | 1393 | 52 | 10 | 0 | 4777 |
|  | Charité | Germany | R | ATATTGCAGCAGTACGCACACA | 26381<–26360 | 4185 | 495296 | 494583 | 711 | 2 | 0 | 0 | 4898 |
| N | CN-CDC | China | F | GGGGAACTTCTCCTGCTAGAAT | 28881–>28902 | 8044 | 492397 | 25524 | 431761 | 2045 | 32107 | 0 | 465913 |
|  | CN-CDC | China | P | TTGCTGCTGCTTGACAGATT | 28934–>28953 | 28051 | 471430 | 467813 | 3617 | 0 | 0 | 0 | 31668 |
|  | CN-CDC | China | R | CAGACATTTTGCTCTCAAGCTG | 28979<–28958 | 35608 | 463873 | 360924 | 13926 | 0 | 0 | 0 | 138557 |
|  | NIH-TH_N | Thailand | F | CGTTTGGTGGACCCTCAGAT | 28320–>28339 | 2824 | 496657 | 490394 | 6263 | 0 | 0 | 0 | 9087 |
|  | NIH-TH_N | Thailand | P | CAACTGGCAGTAACCA | 28341–>28356 | 3063 | 496418 | 495523 | 895 | 0 | 0 | 0 | 3958 |
|  | NIH-TH_N | Thailand | R | CCCCACTGCGTTCTCCATT | 28376<–28358 | 4250 | 495231 | 495179 | 52 | 0 | 0 | 0 | 4302 |
|  | US-CDC-1 | US | F | GACCCCAAAATCAGCGAAAT | 28287–>28306 | 2546 | 496935 | 466654 | 30281 | 0 | 0 | 0 | 32827 |
|  | US-CDC-1 | US | P | ACCCCGCATTACGTTTGGTGGACC | 28309–>28332 | 2874 | 496607 | 489452 | 7148 | 7 | 0 | 0 | 10029 |
|  | US-CDC-1 | US | R | TCTGGTTACTGCCAGTTGAATCTG | 28358<–28335 | 2141 | 497340 | 495311 | 2020 | 9 | 0 | 0 | 4170 |
|  | US-CDC-2 | US | F | TTACAAACATTGGCCGCAAA | 29164–>29183 | 3673 | 495808 | 491768 | 4039 | 1 | 0 | 0 | 7713 |
|  | US-CDC-2 | US | P | ACAATTTGCCCCCAGCGCTTCAG | 29188–>29210 | 6552 | 492929 | 3222 | 8 | 4 | 0 | 0 | 6564 |
|  | US-CDC-2 | US | R | GCGCGACATTCCGAAGAA | 29230<–29213 | 6671 | 492810 | 492707 | 103 | 0 | 0 | 0 | 6774 |
|  | US-CDC-3 | US | F | GGGAGCCTTGAATACACCAAAA | 28681–>28702 | 1860 | 497621 | 494285 | 3336 | 0 | 0 | 0 | 5196 |
|  | US-CDC-3 | US | P | AYCACATTGGCACCCGCAATCCTG | 28704–>28727 | 1530 | 497951 | 496374 | 1563 | 14 | 0 | 0 | 3107 |
|  | US-CDC-3 | US | R | TGTAGCACGATTGCAGCATTG | 28752<–28732 | 2669 | 496812 | 495507 | 1305 | 0 | 0 | 0 | 3974 |
|  | Young | Singapore | F | CTCAGTCCAAGATGGTATTTCT | 28583–>28604 | 3457 | 496024 | 495487 | 537 | 0 | 0 | 0 | 3994 |
|  | Young | Singapore | P | ACCTAGGAACTGGCCCAGAAGCT | 28608–>28630 | 5194 | 494287 | 1 | 494201 | 85 | 0 | 0 | 499480 |
|  | Young | Singapore | R | AGCACCATAGGGAAGTCC | 28648<–28631 | 5156 | 494325 | 494199 | 126 | 0 | 0 | 0 | 5282 |
|  | Corman | Germany | F | CACATTGGCACCCGCAATC | 28706–>28724 | 2886 | 496595 | 496549 | 46 | 0 | 0 | 0 | 2932 |
|  | Corman | Germany | P | ACTTCCTCAAGGAACAACATTGCCA | 28754–>28777 | 3475 | 496006 | 494446 | 1525 | 35 | 0 | 0 | 5035 |
|  | Corman | Germany | R | GAGGAACGAGAAGAGGCTTG | 28833<–28814 | 6108 | 493374 | 491798 | 1575 | 0 | 0 | 0 | 7683 |
|  | Won | South Korea | F | CAATGCTGCAATCGTGCTAC | 28732–>28751 | 2726 | 496755 | 495618 | 1137 | 0 | 0 | 0 | 3863 |
|  | Won | South Korea | R | GTTGCGACTACGTGATGAGG | 28849<–28830 | 6778 | 492703 | 492017 | 686 | 0 | 0 | 0 | 7464 |
|  | NIID-JP | Japan | F | AAATTTTGGGGACCAGGAAC | 29125–>29144 | 2691 | 496790 | 495356 | 1434 | 0 | 0 | 0 | 4125 |
|  | NIID-JP | Japan | P | ATGTCGCGCATTGGCATGGA | 29222–>29241 | 4417 | 495064 | 492738 | 2326 | 0 | 0 | 0 | 6743 |
|  | NIID-JP | Japan | R | TGGCAGCTGTGTAGGTCAAC | 29282<–29263 | 8837 | 490644 | 0 | 490644 | 0 | 0 | 0 | 499481 |
|  | NIID-JP | Japan | R-v3 | TGGCACCTGTGTAGGTCAAC | 29282<–29263 | 3647 | 495834 | 492581 | 3251 | 2 | 0 | 0 | 6900 |
|  | HKU | Hong Kong | F | TAATCAGACAAGGAACTGATTA | 29145–>29166 | 3019 | 496462 | 495278 | 1184 | 0 | 0 | 0 | 4203 |
|  | HKU | Hong Kong | P | GCAAATTGTGCAATTTGCGG | 29177<–29196 | 3285 | 496196 | 495639 | 556 | 1 | 0 | 0 | 3842 |
|  | HKU | Hong Kong | R | CGAAGGTGTGACTTCCATG | 29254<–29236 | 3394 | 496087 | 492187 | 356 | 3544 | 0 | 0 | 7294 |
|  | Chan | China | F | GCGTTCTTCGGAATGTCG | 29210–>29227 | 5838 | 493643 | 493544 | 99 | 0 | 0 | 0 | 5937 |
|  | Chan | China | P | AACGTGGTTGACCTACACAGST | 29257–>29278 | 4157 | 495324 | 491956 | 3367 | 1 | 0 | 0 | 7525 |
|  | Chan | China | R | TTGGATCTTTGTCATCCAATTTG | 29306<–29284 | 4125 | 495356 | 492318 | 3025 | 13 | 0 | 0 | 7163 |

*F: Forward primer; P: Probe; R: Reverse primer.
